# Effects of acceptance and commitment therapy on fear of progression in young and middle-aged patients with breast cancer receiving particle therapy: a randomized controlled trial

**DOI:** 10.3389/fpubh.2026.1920215

**Published:** 2026-09-09

**Authors:** Aoxing Sun, Yu Zhang, Shuman Wang, Fei Qin, Wenjie Xu, Yu Zhu, Hongwei Wan

**Affiliations:** 1 Department of Nursing, Shanghai Proton and Heavy Ion Center, Fudan University Cancer Hospital, Shanghai Key Laboratory of Radiation Oncology, Shanghai, China; 2 Shanghai Engineering Research Center of Proton and Heavy Ion Radiation Therapy, Shanghai, China

**Keywords:** acceptance and commitment therapy, breast cancer, fear of progression, particle therapy, young and middle-aged adults

## Abstract

**Objective:**

Young and middle-aged patients with breast cancer receiving particle therapy commonly experience elevated Fear of Progression (FoP). Evidence for the effectiveness of acceptance and commitment therapy (ACT) in this population remains limited. This study evaluated the effects of ACT on FoP and on related psychological outcomes, namely psychological flexibility and psychological distress.

**Methods:**

This parallel-group randomized controlled trial was conducted at an oncology hospital in Shanghai, China, and enrolled participants between June and August 2025. A total of 108 young and middle-aged patients with breast cancer and elevated FoP (Fear of Progression Questionnaire–Short Form, FoP-Q-SF, ≥34) were randomly assigned in a 1:1 ratio to the intervention group (*n* = 54) or control group (*n* = 54). The intervention group received five group-based sessions (90–120 min each) over 2.5 weeks, covering acceptance, cognitive defusion, present-moment awareness, self-as-context, values clarification, and committed action, whereas the control group received usual care. Outcomes were assessed at baseline, immediately after the intervention, and at 1- and 3-month follow-ups. Intention-to-treat analyses used generalized estimating equations adjusted for cancer stage, age, and education. Sensitivity analyses used multiple imputation.

**Results:**

The overall attrition was 12%, and no intervention-related adverse events were identified. Compared with the control group, the intervention group showed greater reductions in FoP at all post-intervention time points (T1–T3, all *p* < 0.001) and in psychological distress at all follow-ups (T1–T3, all *p* < 0.05). These findings were robust in sensitivity analyses. The effect on psychological flexibility emerged later, reaching statistical significance at T3 (*p* = 0.037) and remaining significant in the sensitivity analysis (*p* = 0.046).

**Conclusion:**

Acceptance and commitment therapy reduced FoP and psychological distress in this population, with a delayed effect on psychological flexibility. These findings support integrating structured, group-based ACT into routine care during particle therapy. Multicenter trials with longer follow-up are needed.

**Trial registration:**

Chinese Clinical Trial Registry Identifier: ChiCTR2500104142.

## 1 Introduction

Breast cancer is a leading cause of cancer-related morbidity and mortality among women worldwide and is increasingly diagnosed in young and middle-aged women (1, 2). The incidence of breast cancer is rising, with a steeper increase among younger women; breast cancer incidence has risen by approximately 1.4% per year in women younger than 50, compared with 0.7% per year in older women (3). Diagnosis during key career and family-building years can disrupt employment, fertility plans, and financial stability (4). To optimize long-term quality of life, an increasing number of these patients receive proton and carbon-ion radiotherapy (collectively, particle therapy). By exploiting the physical Bragg peak, particle therapy deposits a highly localized dose in the target volume while reducing exposure to surrounding organs at risk, such as the heart and lungs, relative to conventional photon therapy (5, 6). Despite this dosimetric precision, patients may still experience treatment-related toxicities, including radiation dermatitis and esophagitis (7, 8).

Patients receiving particle therapy may expect treatment to be both highly precise and free of adverse effects, yet treatment-related toxicities still occur. The gap between expectations and clinical experience may heighten psychological distress (9, 10). Young and middle-aged women, diagnosed at a developmentally critical stage, may be particularly vulnerable (11). When the physical burden of treatment is compounded by anxieties about recurrence, role restoration, and future uncertainty, they become highly susceptible to fear of progression (FoP). Although FoP and fear of cancer recurrence (FCR) have sometimes been used interchangeably (12, 13), the present study focuses specifically on FoP, which emphasizes anticipatory anxiety regarding persistent illness and future uncertainty (14). This focus is particularly relevant to patients undergoing active treatment (15). FoP is among the most prevalent unmet needs of patients with breast cancer and is consistently more severe in younger patients (16, 17). Even a decade after diagnosis, 46% of patients with breast cancer still report high levels of FoP (18). In the absence of timely intervention, such persistent distress may precipitate maladaptive coping behaviors, including avoidance of follow-up examinations and treatment non-adherence, and may be associated with posttraumatic stress and suicidal ideation (19–21). Qualitative evidence further suggests that patients use diverse strategies to manage recurrence-related fear, including seeking support, lifestyle modification, avoidance and emotional detachment, and resilience-building strategies such as acceptance (22), with similar coping patterns also reported in other cancer populations (23). However, FoP among young and middle-aged breast cancer patients receiving particle therapy has rarely been the focus of targeted intervention.

Acceptance and commitment therapy (ACT) is a third-wave behavioral intervention designed to increase psychological flexibility, defined as the ability to contact the present moment fully, embrace internal experiences with an open and accepting attitude, and persist in or change behavior in line with personal values (24). Through acceptance and cognitive defusion, ACT helps patients relate differently to thoughts about disease progression rather than trying to suppress or eliminate them. Value clarification and committed action can help young and middle-aged patients pursue meaningful goals despite uncertainty (25). Several controlled studies support ACT for FoP in breast cancer populations. In a randomized controlled trial of breast cancer patients with high FoP, group-based ACT significantly reduced FoP and anxiety sensitivity while improving psychological flexibility and quality of life relative to a control group (12). A three-arm pilot RCT in breast cancer survivors found that a six-session group ACT intervention produced clinically meaningful and sustained reductions in fear, with effects maintained at follow-up (26). A real-world group program also improved psychological flexibility and quality of life, with further gains between program completion and 12-week follow-up (27). A group format was chosen to provide patients with opportunities to share their experiences, learn from others facing similar concerns, and practice ACT skills in a supportive environment. This approach was also practical during particle therapy, as patients attended the hospital regularly for treatment. However, its therapeutic efficacy in the unique setting of particle therapy has yet to be empirically verified.

Therefore, this randomized controlled trial evaluated whether ACT reduced FoP and improved psychological flexibility and psychological distress in young and middle-aged patients with breast cancer receiving particle therapy. The findings were intended to inform the integration of psychological support into oncology nursing practice.

## 2 Methods

### 2.1 Study design and setting

This single-center, two-arm, parallel-group randomized controlled trial used a 1:1 allocation ratio and was conducted at an oncology hospital in Shanghai, China, from June to August 2025. The institutional ethics committee approved the protocol (Approval No. 2308-67-02-2412A). The study was conducted in accordance with the Declaration of Helsinki and the CONSORT 2025 guidelines. The trial was registered with the Chinese Clinical Trial Registry (Registration No. ChiCTR2500104142).

### 2.2 Participants

Patients were eligible if they (a) were aged 20–59 years (28), (b) had histopathologically confirmed breast cancer according to the 5th edition of the World Health Organization Classification of Tumours of the Breast (29), (c) were currently receiving particle therapy, (d) were aware of their diagnosis, (e) had a clinically significant FoP, defined as a Fear of Progression Questionnaire–Short Form (FoP-Q-SF) score of 34 or higher, and (f) had sufficient literacy and digital literacy to adhere to the intervention. Patients were excluded if they (a) were clinically unstable because of uncontrolled symptoms, acute treatment-related complications, or disease progression requiring urgent medical management, as determined by the treating oncologist, (b) had severe dysfunction of a major organ (heart, lung, liver, or kidney), (c) had a severe mental or cognitive impairment, or (d) were concurrently receiving another psychological intervention or psychotropic medication.

### 2.3 Study procedures

Participants were consecutively recruited from inpatients receiving proton and heavy ion therapy at an oncology hospital in Shanghai, China, between June and August 2025. At admission, a research nurse approached potentially eligible patients, explained the study objectives and procedures, and emphasized that participation was voluntary. After written informed consent was obtained, the research nurse screened each patient against the eligibility criteria, including the FoP-Q-SF. Clinical stability was confirmed by the treating oncologist. Patients who met all criteria (FoP-Q-SF ≥ 34) were enrolled, whereas those who did not were thanked for their willingness to participate. After the baseline assessment (T0), enrolled participants were randomly assigned to the intervention or the control group. Both groups received usual care, and the intervention group additionally received the ACT program. An independent research nurse who was blinded to group allocation collected follow-up data. Outcomes were assessed at baseline (T0), immediately after the intervention (T1), 1 month after the intervention (T2), and 3 months after the intervention (T3).

### 2.4 Randomization, allocation concealment, and blinding

Participants were randomly assigned in a 1:1 ratio using block randomization with a block size of 4. An independent statistician generated the allocation sequence in SPSS version 26.0. The sequence was concealed in sequentially numbered, opaque, sealed envelopes held by independent personnel, and envelopes were opened only after the baseline assessment. Due to the nature of the psychological intervention, participants and therapists could not be blinded; participants were instructed not to disclose their assignment to assessors. Outcome assessors were blinded to group allocation. Although the lead analyst was not blinded, all statistical analyses adhered to a prespecified analysis plan documented in the trial registration and protocol, minimizing the risk of analysis bias.

### 2.5 Control group

Both groups received usual care throughout the study period. Usual care comprised four components. (a) Participants received a health education handbook covering the treatment modality and management of common complications and adverse reactions. (b) Educational videos addressed diet and physical activity, supplemented by dietary, activity, and psychological guidance from the research team. (c) Routine psychosocial support included attentive listening, communication, and emotional reassurance. (d) After discharge, the research team remained available to address patients’ questions in a timely manner.

### 2.6 Intervention group

The standardized protocol, grounded in ACT and tailored to this population (24, 30), was refined through expert consultation, pilot testing, and qualitative interviews with patients. The program comprised five sessions (90–120 min each) delivered twice weekly over 2.5 weeks in closed groups of 6–10 participants. To support attendance, participants received an in-person reminder approximately 2 h before each session. Whenever possible, sessions were scheduled to avoid conflicts with treatment appointments. The five sessions progressed sequentially through the core ACT processes. Session 1 introduced the ACT model and group principles and promoted acceptance of cancer-related distress. Session 2 used cognitive defusion to reduce the influence of progression-related thoughts. Session 3 cultivated present-moment awareness. Session 4 developed self-as-context by helping patients observe thoughts and feelings as transient events. Session 5 focused on values clarification and committed action and guided patients in setting value-based behavioral goals. Each session combined metaphors, experiential exercises, and mindfulness training. Sessions were held in a private, quiet counseling room in the hospital. The prespecified adherence criterion was attendance at three or more sessions (≥60% of the full program). A senior group psychologist served as the lead therapist, and the researcher, an oncology nurse trained in ACT, served as co-therapist. Table 1 provides the detailed intervention protocol.

**Table 1:** The acceptance and commitment therapy intervention protocol.

Session	Core ACT process	Objectives	Key techniques and exercises
1	Acceptance and Group Cohesion	To introduce the ACT model and group principles. To normalize and build acceptance of cancer-related distress (e.g., Fear of Progression).	Metaphor: “T-Puzzle” (on uncontrollability); Experiential Exercise: “Tug-of-War with a Monster” (coexisting with difficult feelings); Mindfulness: Mindful Breathing
2	Cognitive Defusion	To differentiate thoughts from the “self.” To reduce the impact and control of FoP-related thoughts	Metaphor: “Lemon, Lemon” (observing vs. believing thoughts); Experiential Exercise: “Hands as Thoughts” (creating distance); Mindfulness: “Leaves on a Stream” meditation.
3	Contact with the Present Moment	To anchor attention to the present moment. To disengage from past regrets or future worries (e.g., Fear of Progression).	Metaphor: “Sand Painting Art” (impermanence, focus on ‘now’). Experiential Exercise: Mindful observation (e.g., “Observe Your Hand”); Mindfulness: Mindful Raisin Eating.
4	Self-as-Context	To foster a transcendent sense of self (the “observer”). To observe thoughts/feelings as passing events without being defined by them.	Metaphor: “Labeling” (thoughts as “just thoughts”). Experiential Exercise: “Life Curve” (observing one’s life story); Mindfulness: “Mountain Meditation.”
5	Values Clarification and Committed Action	To identify core personal values postdiagnosis. To develop concrete, value-guided behavioral goals and review session learnings.	Metaphor: “The Compass” (values as life direction). Experiential Exercise: “Values Compass” (clarifying values); Group review. Homework: Develop a 3-month SMART plan aligned with values

ACT, acceptance and commitment therapy; FoP, fear of progression; SMART, specific, measurable, achievable, relevant, and time-bound.

### 2.7 Outcome measures

The primary outcome was FoP, assessed with the FoP-Q-SF. Secondary outcomes were psychological flexibility and psychological distress. All outcome variables were assessed at four time points: T0, T1, T2, and T3.

#### 2.7.1 Fear of progression

The primary outcome was assessed via the FoP-Q-SF (31). The scale comprises 12 items rated on a 5-point Likert scale. The total scores range from 12 to 60, with higher scores indicating higher levels of Fear of Progression. A score of ≥ 34 indicates a clinically significant (dysfunctional) level of fear, suggesting a need for psychological intervention. We used the culturally adapted Chinese version, which was validated in 678 Chinese patients with primary liver cancer and demonstrated good reliability and validity, including internal consistency (Cronbach’s α = 0.883) and item-total correlations of 0.587–0.712 (32).

#### 2.7.2 Psychological flexibility

Psychological flexibility was assessed via the Acceptance and Action Questionnaire-II (AAQ-II) (33). The AAQ-II consists of 7 items rated on a 7-point Likert scale, with total scores ranging from 7 to 49. The scale primarily measures experiential avoidance; thus, a higher score indicates greater psychological inflexibility/avoidance, corresponding to lower levels of psychological flexibility. The Chinese AAQ-II has demonstrated acceptable construct and criterion-related validity and good reliability in Chinese college students (Cronbach’s *α* = 0.88; test–retest *r* = 0.80) (34); however, validation in oncology populations remains limited.

#### 2.7.3 Psychological distress

Psychological distress was assessed via the Distress Thermometer (DT) (35). This is a single-item visual analog scale ranging from 0 to 10, where 0 indicates ‘no distress’ and 10 indicates ‘extreme distress.’ Higher scores reflect greater levels of distress. A score of ≥4 has been established as the cutoff threshold for clinically significant distress (36). In the present study, the DT rating scale was administered without the accompanying Problem List. In Chinese cancer patients, the DT demonstrated acceptable criterion validity against the Hospital Anxiety and Depression Scale and the Symptom Checklist-90 (AUC = 0.803 and 0.834, respectively), as well as acceptable test–retest reliability (*r* = 0.80) (36).

### 2.8 Statistical methods

#### 2.8.1 Sample size calculation

Sample size estimation was based on the primary outcome, Fear of Progression. The required sample size per group for comparing two independent means was calculated using the formula *n* = 2(*Z*_1-α/2_ + *Z*_1-β_)^2^/*d*^2^. The assumptions were a two-sided significance level of *α* = 0.05 (*Z*_1-α/2_ = 1.96), a statistical power of 80% (*β* = 0.20, *Z*_1-β_ = 0.84), and a 1:1 allocation ratio between the intervention and control groups. On the basis of a previous ACT study, we anticipated an effect size (d) of 0.61 (26). The calculation yielded 42.15 participants per group, which was rounded up to 43. After allowing for 20% attrition (43/0.80 = 53.75), the required sample size was rounded up to 54 participants per group, resulting in a total target sample of 108 participants.

#### 2.8.2 Data analysis

Analyses followed the intention-to-treat (ITT) principle and were conducted in R version 4.5.1. Continuous variables were assessed for normality using the Shapiro–Wilk test and are reported as means ± SDs or medians (IQRs), as appropriate. Categorical variables are reported as *n* (%). Baseline differences were assessed using *t*-tests, Mann–Whitney *U* tests, chi-square tests, or Fisher’s exact tests, as appropriate. Patterns of missing data were examined by comparing baseline characteristics between completers and participants lost to follow-up.

Longitudinal effects were estimated using Generalized Estimating Equations (GEEs) with a Gaussian distribution, identity link, exchangeable working correlation structure, and robust standard errors. Models included group, time, and group × time interaction terms. Baseline variables that differed between groups or were associated with loss to follow-up were included as covariates. Group × time interactions were the primary measures of intervention effects. Estimated marginal means (EMMs) with 95% CIs were calculated to describe the outcome trajectories. The Holm method adjusted within-group pairwise comparisons. Statistical significance was defined as a two-sided *p* < 0.05.

To assess the robustness of the findings to departures from the missing-completely-at-random (MCAR) assumption (37), sensitivity analyses used multiple imputation. The imputation model included treatment group, covariates, outcomes, and auxiliary variables. Twenty imputed datasets were generated by predictive mean matching with the R package mice, and estimates were pooled using Rubin’s rules.

#### 2.8.3 Treatment fidelity

All interventions followed a standardized manual and were delivered by therapists who had completed systematic ACT training. Every session was audio-recorded. A clinical supervisor reviewed a random 20% sample of recordings with an adherence checklist, and weekly supervision meetings were used to address any protocol deviations.

## 3 Results

### 3.1 Recruitment

**Figure 1:**
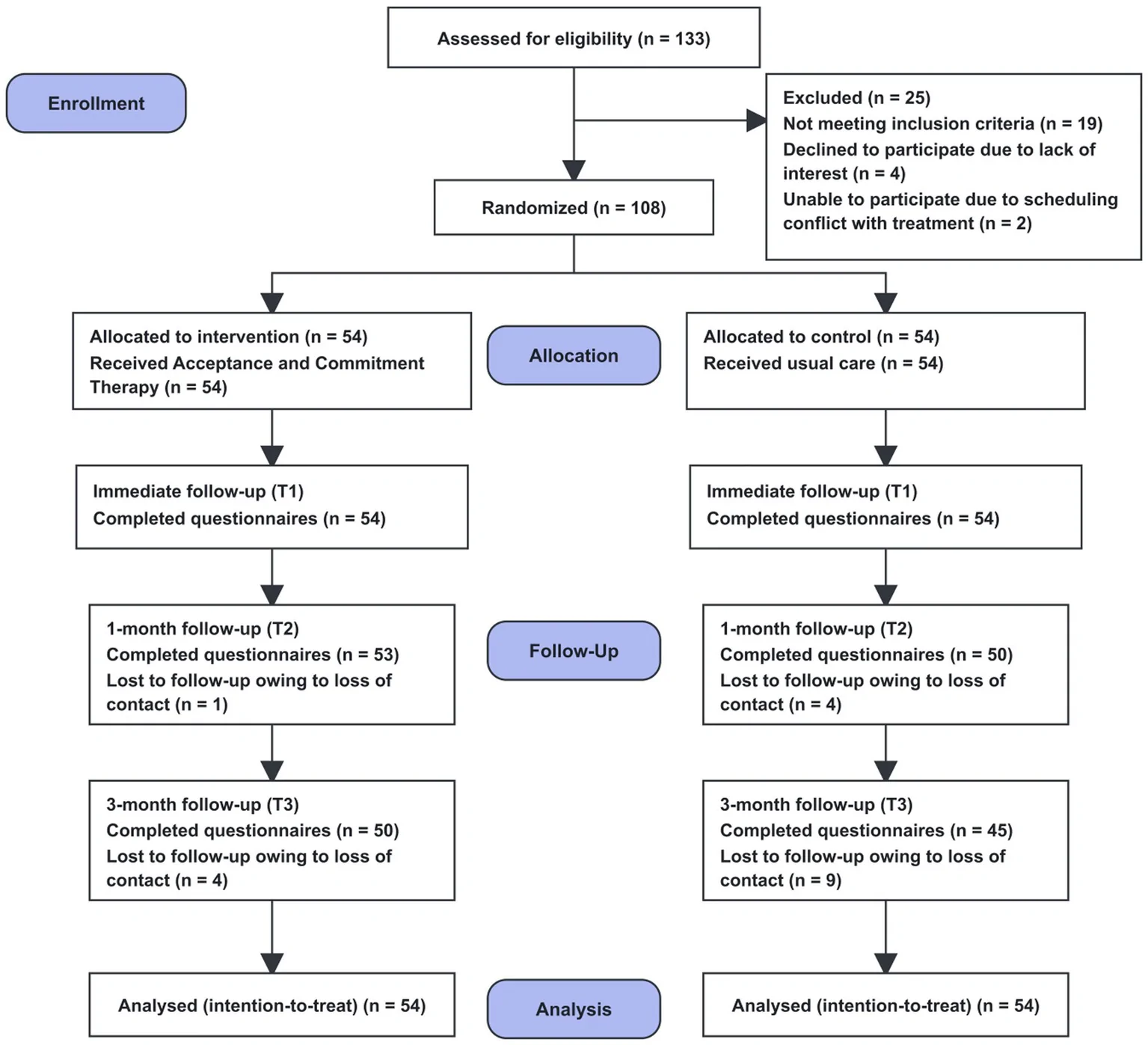
CONSORT flow diagram of participant recruitment, randomization, follow-up, and analysis.

A total of 133 patients were assessed for eligibility, of whom 25 were excluded for the following reasons: failure to meet the inclusion criteria (*n* = 19), lack of interest (*n* = 4), and time conflicts (*n* = 2). The remaining 108 eligible patients were randomly assigned to the intervention group (*n* = 54) or the control group (*n* = 54). At T2, 1 participant in the intervention group and 4 in the control group were lost to follow-up, and a further 3 and 5 participants were lost in the two groups, respectively, by T3. In total, 50 participants in the intervention group and 45 in the control group completed the study, corresponding to attrition rates of 7.4% (4/54) and 16.7% (9/54), respectively, and an overall attrition rate of 12.0% (13/108). The recruitment and follow-up process is detailed in Figure 1.

### 3.2 Baseline characteristics

The mean age was 43.43 ± 8.46 years in the intervention group and 42.37 ± 8.08 years in the control group. At baseline, the median FoP-Q-SF score was 36 (IQR 35–38) in the intervention group and 37 (IQR 36–39) in the control group. Both values exceeded the clinical cutoff of 34. Psychological flexibility and psychological distress scores were similar between groups at baseline. Baseline characteristics were balanced except for cancer stage (*p* = 0.029). Stage III disease was more common in the intervention group than in the control group (42.6% vs. 24.1%), whereas stage II disease was more common in the control group (55.6% vs. 31.5%). Cancer stage was therefore included as a covariate in all subsequent GEE analyses. Table 2 presents the baseline characteristics.

**Table 2:** Baseline characteristics of the two groups (*N* = 108).

Characteristics	Control group	Intervention group	Total (*N* = 108)	Statistic	*P*
	(*n* = 54)	(*n* = 54)			
Age (years)	42.37 ± 8.08	43.43 ± 8.46	42.90 ± 8.25	−0.775^1^	0.441
Monthly *Per Capita* Household Income (CNY)				5.103^2^	0.164
<10 k	19 (35.2%)	10 (18.5%)	29 (26.9%)		
(10 k, 20 k)	16 (29.6%)	21 (38.9%)	37 (34.3%)		
(20 k, 30 k)	10 (18.5%)	16 (29.6%)	26 (24.1%)		
≥30 k	9 (16.7%)	7 (13.0%)	16 (14.8%)		
Time since initial diagnosis (months)				1.971^2^	0.373
(3, 6)	5 (9.3%)	9 (17%)	14 (13%)		
(6, 12)	45 (83%)	39 (72%)	84 (78%)		
≥12	4 (7.4%)	6 (11%)	10 (9.3%)		
Marital Status				1.296^3^	0.761
Single/Unmarried	5 (9.3%)	7 (13.0%)	12 (11.1%)		
Married	48 (88.9%)	47 (87.0%)	95 (88.0%)		
Divorced	1 (1.9%)	0 (0%)	1 (0.9%)		
Employment Status				4.117^2^	0.128
Permanent Employment	40 (74%)	30 (56%)	70 (65%)		
Freelance	7 (13%)	13 (24%)	20 (19%)		
Retired	7 (13%)	11 (20%)	18 (17%)		
Education Level				2.102^3^	0.609
Primary school or below	2 (3.7%)	0 (0%)	2 (1.9%)		
Secondary school	14 (25.9%)	17 (31.5%)	31 (28.7%)		
University	32 (59.3%)	30 (55.6%)	62 (57.4%)		
Master’s degree or above	6 (11.1%)	7 (13.0%)	13 (12.0%)		
Religious Affiliation				3.215^3^	0.314
None	45 (83.3%)	50 (92.6%)	95 (88.0%)		
Christianity	2 (3.7%)	2 (3.7%)	4 (3.7%)		
Buddhism	6 (11.1%)	2 (3.7%)	8 (7.4%)		
Islam	1 (1.9%)	0 (0%)	1 (0.9%)		
Medical Insurance				2.550^2^	0.110
No	9 (17%)	16 (30%)	25 (23%)		
Yes	45 (83%)	38 (70%)	83 (77%)		
Primary Caregiver				4.287^3^	0.367
Spouse	35 (64.8%)	33 (61.1%)	68 (63.0%)		
Parents	7 (13.0%)	12 (22.2%)	19 (17.6%)		
Children	3 (5.6%)	5 (9.3%)	8 (7.4%)		
Paid Caregiver	1 (1.9%)	1 (1.9%)	2 (1.9%)		
Others	8 (14.8%)	3 (5.6%)	11 (10.2%)		
Treatment Regimen				0.532^2^	0.466
Proton Therapy	45 (83%)	42 (78%)	87 (81%)		
Proton and Heavy Ion Therapy	9 (17%)	12 (22%)	21 (19%)		
Cancer Stage				9.080^3^	0.029
0	1 (1.9%)	0 (0%)	1 (0.9%)		
I	10 (18.5%)	12 (22.2%)	22 (20.4%)		
II	30 (55.6%)	17 (31.5%)	47 (43.5%)		
III	13 (24.1%)	23 (42.6%)	36 (33.3%)		
IV	0 (0%)	2 (3.7%)	2 (1.9%)		
Molecular Subtype				3.288^3^	0.352
Luminal A	2 (3.7%)	5 (9.3%)	7 (6.5%)		
Luminal B	40 (74.1%)	41 (75.9%)	81 (75.0%)		
HER2-enriched	3 (5.6%)	4 (7.4%)	7 (6.5%)		
Triple-negative	9 (16.7%)	4 (7.4%)	13 (12.0%)		

Values are *M* ± SD or *n* (%), as appropriate. CNY, Chinese yuan. ^1^Student’s *t*-test. ^2^Chi-square test. ^3^Fisher’s exact test.

### 3.3 Missing data analysis

Among the 13 participants lost to follow-up, attrition rates did not differ significantly between groups at T2 (*p* = 0.363) or T3 (*p* = 0.237), as shown in Table 3. Compared with completers (n = 95), participants lost to follow-up (*n* = 13) were older (*p* = 0.026) and had lower educational attainment (*p* = 0.006). No other baseline demographic, clinical, or outcome variables differed significantly (Table 4). Under the prespecified covariate-selection criteria, age and education were therefore included as additional covariates in subsequent GEE analyses.

**Table 3:** Data completeness and between-group attrition rates at each time point.

Time point	Control group, valid cases (missing)	Intervention group, valid cases (missing)	Overall attrition rate	Statistic	*P*
T0	54 (0, 0%)	54 (0, 0%)	0%	–	–
T1	54 (0, 0%)	54 (0, 0%)	0%	–	–
T2	50 (4, 7.4%)	53 (1, 1.9%)	4.6%	–	0.363^1^
T3	45 (9, 16.7%)	50 (4, 7.4%)	12.0%	1.399	0.237^2^

Values are *n* (missing, missing percentage), unless otherwise indicated. ^1^Fisher’s exact test. ^2^Chi-square test with continuity correction.

**Table 4:** Baseline characteristics of completers and participants lost to follow-up.

Characteristics	Completers (*n* = 95)	Non-completers (*n* = 13)	Statistic	*P*
Age (years)	41 (37, 47)	48 (45, 50)	−2.231^1^	0.026
Monthly *Per Capita* Household Income (CNY)				0.109^3^
<10 k	22 (23.2%)	7 (53.8%)		
(10 k, 20 k)	33 (34.7%)	4 (30.8%)		
(20 k, 30 k)	24 (25.3%)	2 (15.4%)		
≥30 k	16 (16.8%)	0 (0%)		
Time since initial diagnosis (months)				1.000^3^
(3, 6)	13 (13.7%)	1 (7.7%)		
(6, 12)	73 (76.8%)	11 (84.6%)		
≥12	9 (9.5%)	1 (7.7%)		
Marital Status				0.154^3^
Single/Unmarried	11 (11.6%)	1 (7.7%)		
Married	84 (88.4%)	11 (84.6%)		
Divorced	0 (0%)	1 (7.7%)		
Employment Status				0.828^3^
Permanent Employment	62 (65.3%)	8 (61.5%)		
Freelance	18 (18.9%)	2 (15.4%)		
Retired	15 (15.8%)	3 (23.1%)		
Education Level				0.006^3^
Primary school or below	0 (0%)	2 (15.4%)		
Secondary school	25 (26.3%)	6 (46.2%)		
University	58 (61.1%)	4 (30.8%)		
Master’s degree or above	12 (12.6%)	1 (7.7%)		
Religious affiliation				0.285^3^
None	85 (89.5%)	10 (76.9%)		
Christianity	3 (3.2%)	1 (7.7%)		
Buddhism	6 (6.3%)	2 (15.4%)		
Islam	1 (1.1%)	0 (0%)		
Medical Insurance				1.000^3^
No	22 (23.2%)	3 (23.1%)		
Yes	73 (76.8%)	10 (76.9%)		
Primary Caregiver				0.243^3^
Spouse	60 (63.2%)	8 (61.5%)		
Parents	18 (18.9%)	1 (7.7%)		
Children	5 (5.3%)	3 (23.1%)		
Paid Caregiver	2 (2.1%)	0 (0%)		
Others	10 (10.5%)	1 (7.7%)		
Treatment Regimen			1.210^2^	0.271
Proton Therapy	78 (82.1%)	9 (69.2%)		
Proton and Heavy Ion Therapy	17 (17.9%)	4 (30.8%)		
Cancer Stage				0.426^3^
0	1 (1.1%)	0 (0%)		
I	19 (20.0%)	3 (23.1%)		
II	41 (43.2%)	6 (46.2%)		
III	33 (34.7%)	3 (23.1%)		
IV	1 (1.1%)	1 (7.7%)		
Molecular Subtype				0.441^3^
Luminal A	6 (6.3%)	1 (7.7%)		
Luminal B	72 (75.8%)	9 (69.2%)		
HER2-enriched	5 (5.3%)	2 (15.4%)		
Triple-negative	12 (12.6%)	1 (7.7%)		
Group Allocation, *n* (%)			2.186^2^	0.139
Control	45 (47.4%)	9 (69.2%)		
ACT	50 (52.6%)	4 (30.8%)		
Baseline Outcomes				
Fear of Progression, Median (IQR)	37 (35, 38)	36 (36, 39)	−0.325^1^	0.745
Psychological Flexibility, M ± SD	28.08 ± 4.79	26.85 ± 5.55	0.858^4^	0.393
Psychological Distress, Median (IQR)	3 (1, 3)	3 (2, 3)	−0.876^1^	0.381

Values are median (IQR), *M* ± SD, or *n* (%), as appropriate. ACT, acceptance and commitment therapy; IQR, interquartile range; SD, standard deviation. ^1^Mann–Whitney *U* test (*Z* statistic). ^2^Chi-square test. ^3^Fisher’s exact test. ^4^Independent-samples *t*-test.

### 3.4 Intervention adherence

Intervention adherence was assessed by recording session attendance. In the intervention group, mean attendance was 3.89 sessions. Forty-nine participants (90.7%) met the prespecified adherence criterion of attending at least three sessions, including 19 (35.2%) who completed all five sessions. Five participants (9.3%) attended fewer than three sessions. Reasons for missed sessions were not systematically recorded.

### 3.5 Primary outcomes

The GEE analysis (Table 5) showed no significant difference in baseline FoP (*β* = −0.38, *p* = 0.389). Group × Time interactions were significant at all post-intervention assessments (T1: *β* = −4.63, *p* < 0.001; T2: *β* = −4.13, *p* < 0.001; T3: *β* = −3.11, *p* < 0.001), indicating a greater reduction in FoP in the intervention group than in the control group.

**Table 5:** Generalized estimating equation analyses of outcome measures.

Outcomes	Time	Intervention group (raw scores)	Control group (raw scores)	Group effect	Time effect	Interaction effect
		M (SD)/Median (IQR)	M (SD)/Median (IQR)	*β* (95% CI), *P*	*β* (95% CI), *P*	*β* (95% CI), *P*
Fear of Progression (FoP-Q-SF)	T0	36 (35, 38)	37 (36, 39)	−0.38 (−1.23, 0.48), 0.389		
	T1	30.41 ± 4.17	35.5 ± 2.93		−1.74 (−2.64, −0.84), <0.001	−4.63 (−6.23, −3.03), <0.001
	T2	29.60 ± 3.69	34.22 ± 3.99		−3.03 (−4.13, −1.94), <0.001	−4.13 (−5.65, −2.61), <0.001
	T3	28.54 ± 3.55	32.09 ± 4.01		−5.11 (−6.46, −3.76), <0.001	−3.11 (−4.84, −1.38), <0.001
Psychological Flexibility (AAQ-II)	T0	27.35 ± 4.77	28.52 ± 4.96	−1.29 (−3.12, 0.53), 0.165		
	T1	22.87 ± 5.98	25.59 ± 5.66		−2.93 (−4.66, −1.19), <0.001	−1.37 (−4.03, 1.29), 0.313
	T2	21 (17, 25)	24.78 ± 3.87		−3.72 (−5.43, −2.02), <0.001	−2.59 (−5.22, 0.03), 0.053
	T3	20.08 ± 5.95	24.29 ± 5.85		−4.22 (−6.45, −1.98), <0.001	−3.20 (−6.22, −0.19), 0.037
Psychological Distress (DT)	T0	3 (1, 3)	3 (2, 3)	−0.41 (−0.84, 0.02), 0.059		
	T1	2 (1, 2)	2 (2, 3)		−0.15 (−0.24, −0.05), 0.002	−0.32 (−0.52, −0.11), 0.003
	T2	1 (1, 2)	2 (2, 3)		−0.38 (−0.51, −0.25), <0.001	−0.33 (−0.58, −0.07), 0.012
	T3	1 (1, 1.25)	2 (1, 2)		−0.51 (−0.65, −0.37), <0.001	−0.52 (−0.81, −0.23), <0.001

Raw scores are presented as *M* ± SD or median (IQR), as appropriate. AAQ-II, Acceptance and Action Questionnaire-II; CI, confidence interval; DT, Distress Thermometer; FoP-Q-SF, Fear of Progression Questionnaire-Short Form; GEE, generalized estimating equation; IQR, interquartile range; SD, standard deviation. All models were adjusted for cancer stage, age, and education level and used robust (sandwich) standard errors. The group effect represents the between-group difference at T0; the time effect represents change in the control group relative to T0; and the interaction effect represents the additional change in the intervention group relative to the control group.

**Figure 2:**
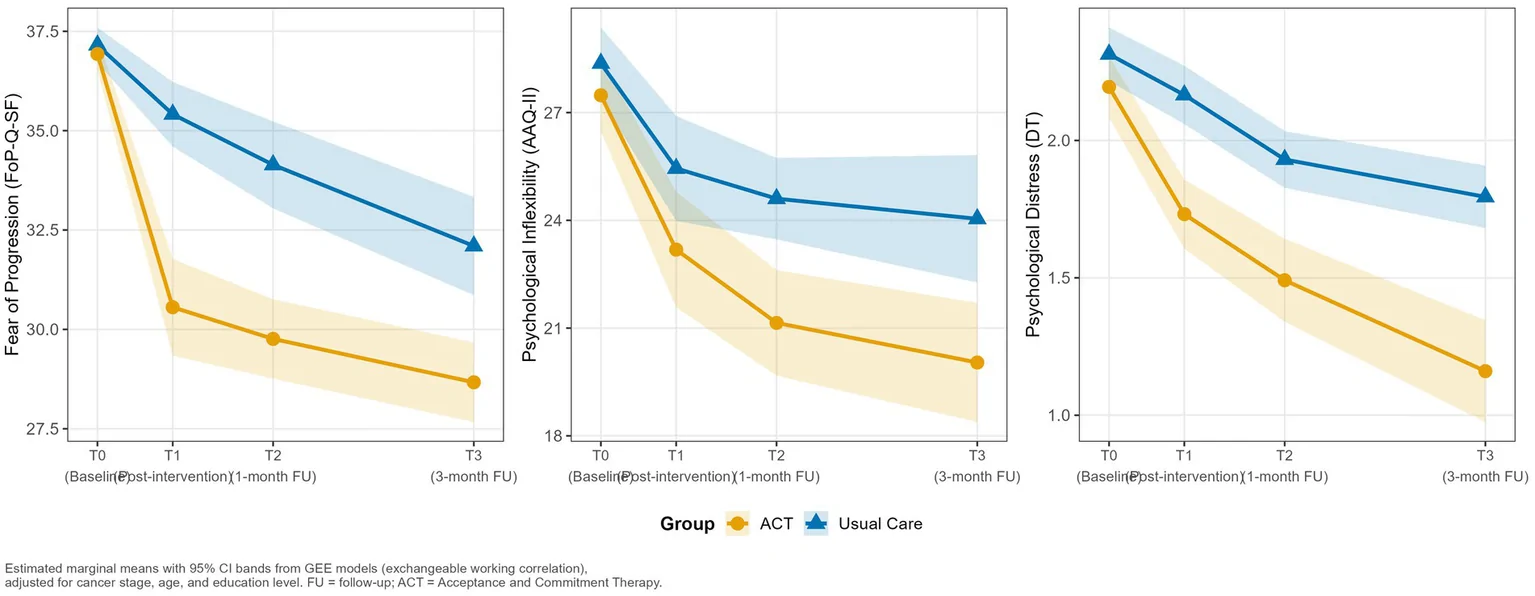
Estimated marginal mean trajectories of fear of progression, psychological flexibility, and psychological distress in both groups over time.

Pairwise comparisons of estimated marginal means (Table 6) showed lower FoP scores in the intervention group than in the control group at T1 (MD = −5.01, *p* < 0.001), T2 (MD = −4.50, *p* < 0.001), and T3 (MD = −3.49, *p* < 0.001). In the intervention group, the largest reduction occurred between baseline and T1 (MD = −6.37, *p* < 0.001), with further improvement through T3 (T1 vs. T3: MD = −1.85, *p* = 0.033). The control group also improved over time (T0 vs. T3: MD = −5.11, *p* < 0.001), although the change was more gradual. Table 7 presents the estimated marginal means at each assessment, and Figure 2 shows the trajectories of both groups.

**Table 6:** Pairwise comparisons of estimated marginal means for fear of progression.

Comparison	Time	MD	SE	95% CI	*P*
Intervention Group (within-group)	T1–T0	−6.37	0.674	[−8.16, −4.58]	<0.001
	T2–T0	−7.16	0.541	[−8.60, −5.73]	<0.001
	T3–T0	−8.22	0.552	[−9.68, −6.76]	<0.001
	T2–T1	−0.79	0.794	[−2.90, 1.30]	0.319
	T3–T1	−1.85	0.766	[−3.88, 0.18]	0.033
	T3–T2	−1.06	0.177	[−1.53, −0.59]	<0.001
Control Group (Within-group)	T1–T0	−1.74	0.459	[−2.96, −0.52]	<0.001
	T2–T0	−3.04	0.558	[−4.51, −1.56]	<0.001
	T3–T0	−5.11	0.688	[−6.93, −3.28]	<0.001
	T2–T1	−1.29	0.621	[−2.94, 0.35]	0.038
	T3–T1	−3.37	0.727	[−5.29, −1.44]	<0.001
	T3–T2	−2.07	0.853	[−4.33, 0.19]	0.031
Intervention–Control (Between-group)	T0	−0.38	0.437	[−1.23, 0.48]	0.389
	T1	−5.01	0.696	[−6.38, −3.64]	<0.001
	T2	−4.50	0.749	[−5.98, −3.03]	<0.001
	T3	−3.49	0.782	[−5.03, −1.95]	<0.001

CI, confidence interval; EMM, estimated marginal mean; GEE, generalized estimating equation; MD, mean difference; SE, standard error. Within-group comparisons represent change over time; between-group comparisons represent intervention minus control at each time point.

**Table 7:** Estimated marginal means and 95% confidence intervals for outcome measures.

Outcomes	Group	T0 EMM	95% CI	T1 EMM	95% CI	T2 EMM	95% CI	T3 EMM	95% CI
Fear of Progression	Control	37.23	(36.52, 37.94)	35.48	(34.63, 36.34)	34.19	(33.03, 35.36)	32.12	(30.89, 33.35)
	Intervention	36.85	(36.06, 37.64)	30.48	(29.34, 31.62)	29.69	(28.62, 30.75)	28.63	(27.59, 29.68)
Psychological Flexibility	Control	28.41	(26.98, 29.84)	25.49	(23.86, 27.11)	24.69	(23.51, 25.86)	24.20	(22.43, 25.96)
	Intervention	27.12	(25.81, 28.44)	22.82	(21.11, 24.54)	20.80	(19.25, 22.35)	19.70	(18.09, 21.31)
Psychological Distress	Control	2.49	(2.17, 2.81)	2.34	(2.04, 2.64)	2.11	(1.85, 2.37)	1.98	(1.72, 2.25)
	Intervention	2.08	(1.70, 2.45)	1.61	(1.32, 1.91)	1.37	(1.10, 1.65)	1.05	(0.77, 1.32)

CI, confidence interval; EMM, estimated marginal mean; GEE, generalized estimating equation. Models were adjusted for cancer stage, age, and education level, with an exchangeable working correlation structure and robust standard errors.

### 3.6 Secondary outcomes

For Psychological flexibility, the group × time interaction was not significant at T1 (*β* = −1.37, *p* = 0.313), was of borderline significance at T2 (*β* = −2.59, *p* = 0.053), and was significant at T3 (*β* = −3.20, *p* = 0.037). Pairwise comparisons (Table 8) showed that the between-group mean difference increased over time and reached MD = −4.49 at T3 (*p* < 0.001). Within the intervention group, AAQ-II scores decreased significantly from baseline to T3 (MD = −7.42, *p* < 0.001), compared with a smaller decrease in the control group (MD = −4.22, *p* = 0.001).

**Table 8:** Pairwise comparisons of estimated marginal means for psychological flexibility.

Comparison	Time	MD	SE	95% CI	*P*
Intervention Group (Within-group)	T1–T0	−4.30	1.03	[−7.03, −1.57]	<0.001
	T2–T0	−6.32	1.02	[−9.02, −3.61]	<0.001
	T3–T0	−7.42	1.04	[−10.17, −4.67]	<0.001
	T2–T1	−2.02	1.11	[−4.95, 0.91]	0.136
	T3–T1	−3.12	1.22	[−6.37, 0.12]	0.033
	T3–T2	−1.10	1.11	[−4.05, 1.85]	0.322
Control Group (Within-group)	T1–T0	−2.93	0.89	[−5.28, −0.57]	0.004
	T2–T0	−3.73	0.87	[−6.04, −1.42]	<0.001
	T3–T0	−4.22	1.14	[−7.24, −1.20]	0.001
	T2–T1	−0.80	0.89	[−3.14, 1.55]	0.733
	T3–T1	−1.29	1.06	[−4.11, 1.53]	0.676
	T3–T2	−0.49	0.92	[−2.93, 1.94]	0.733
Intervention–Control (Between-group)	T0	−1.29	0.93	[−3.12, 0.53]	0.166
	T1	−2.66	1.12	[−4.87, −0.46]	0.018
	T2	−3.89	0.89	[−5.63, −2.14]	<0.001
	T3	−4.49	1.17	[−6.80, −2.94]	<0.001

CI, confidence interval; EMM, estimated marginal mean; GEE, generalized estimating equation; MD, mean difference; SE, standard error. Within-group comparisons represent change over time; between-group comparisons represent intervention minus control at each time point.

For psychological distress, group × time interactions were significant at all post-intervention assessments (T1: *β* = −0.32, *p* = 0.003; T2: *β* = −0.33, *p* = 0.012; T3: *β* = −0.52, *p* < 0.001). Pairwise comparisons (Table 9) showed consistently lower distress scores in the intervention group than in the control group, with the largest between-group difference at T3 (MD = −0.93, *p* < 0.001). Distress scores decreased from baseline to T3 in both groups, with a larger decrease in the intervention group (MD = −1.03, *p* < 0.001), than in the control group (MD = −0.51, *p* < 0.001). Figure 2 shows the estimated marginal mean trajectories for both secondary outcomes.

**Table 9:** Pairwise comparisons of estimated marginal means for psychological distress.

Comparison	Time	MD	SE	95% CI	*P*
Intervention Group (Within-group)	T1–T0	−0.46	0.09	[−0.71, −0.22]	<0.001
	T2–T0	−0.70	0.11	[−1.00, −0.41]	<0.001
	T3–T0	−1.03	0.13	[−1.37, −0.69]	<0.001
	T2–T1	−0.24	0.08	[−0.44, −0.04]	0.001
	T3–T1	−0.57	0.08	[−0.78, −0.36]	<0.001
	T3–T2	−0.33	0.07	[−0.50, −0.15]	<0.001
Control Group (Within-group)	T1–T0	−0.15	0.05	[−0.28, −0.02]	0.005
	T2–T0	−0.38	0.07	[−0.56, −0.20]	<0.001
	T3–T0	−0.51	0.07	[−0.70, −0.32]	<0.001
	T2–T1	−0.23	0.06	[−0.38, −0.08]	<0.001
	T3–T1	−0.36	0.07	[−0.54, −0.18]	<0.001
	T3–T2	−0.13	0.05	[−0.27, 0.003]	0.01
Intervention–Control (Between-group)	T0	−0.41	0.22	[−0.84, 0.02]	0.06
	T1	−0.73	0.18	[−1.08, −0.38]	<0.001
	T2	−0.74	0.15	[−1.04, −0.44]	<0.001
	T3	−0.93	0.16	[−1.24, −0.63]	<0.001

CI, confidence interval; EMM, estimated marginal mean; GEE, generalized estimating equation; MD, mean difference; SE, standard error. Within-group comparisons represent change over time; between-group comparisons represent intervention minus control at each time point.

### 3.7 Sensitivity analysis

Sensitivity analyses using multiple imputation are presented in Table 10. For FoP, group × time interactions remained significant at all post-baseline assessments (T1: *β* = −4.63, *p* < 0.001; T2:*β* = −4.11, *p* < 0.001; T3: *β* = −2.94, *p* = 0.020). Results for psychological distress were also consistent with the primary analysis (T1: *p* = 0.003; T2: *p* = 0.016; T3: *p* = 0.002). For psychological flexibility, the interaction at T3 remained significant (*β* = −3.18, *p* = 0.046), while the earlier assessments showed the same non-significant pattern as the primary analysis (T1: *p* = 0.314; T2: *p* = 0.059). Overall, the consistency between the main and imputed analyses indicates that the observed intervention effects are not attributable to the missing data mechanism.

**Table 10:** Sensitivity analysis comparing Generalized Estimating Equation coefficients before and after Multiple Imputation.

Outcomes	Time	Main analysis						Multiple imputation					
		Group		Time		Interaction		Group		Time		Interaction	
		*β*	*P*	*β*	*P*	*β*	*P*	*β*	*P*	*β*	*P*	*β*	*P*
Fear of Progression	T0	−0.38	0.389					−0.37	0.398				
	T1			−1.74	<0.001	−4.63	<0.001			−1.74	<0.001	−4.63	<0.001
	T2			−3.03	<0.001	−4.13	<0.001			−3.03	<0.001	−4.11	<0.001
	T3			−5.11	<0.001	−3.11	<0.001			−5.26	<0.001	−2.94	0.020
Psychological Flexibility	T0	−1.29	0.165					−1.28	0.169				
	T1			−2.93	<0.001	−1.37	0.313			−2.93	0.001	−1.37	0.314
	T2			−3.73	<0.001	−2.59	0.053			−3.72	<0.001	−2.55	0.059
	T3			−4.22	<0.001	−3.20	0.037			−4.18	<0.001	−3.18	0.046
Psychological Distress	T0	−0.41	0.059					−0.41	0.060				
	T1			−0.15	0.002	−0.32	0.003			−0.15	0.002	−0.32	0.003
	T2			−0.38	<0.001	−0.33	0.012			−0.39	0.033	−0.32	0.016
	T3			−0.51	<0.001	−0.52	<0.001			−0.55	0.106	−0.47	0.002

GEE, generalized estimating equation. All models were adjusted for cancer stage, age, and education level. The group effect represents the between-group difference at T0; the time effect represents change in the control group relative to T0; and the interaction effect represents the additional change in the intervention group relative to the control group.

### 3.8 Harms

No deaths occurred during the trial period, and no participants withdrew because of intervention-related harms. No intervention-related adverse events were identified through session audio-recording review or weekly clinical supervision during the intervention period. Participants continued to receive routine clinical care and monitoring during particle therapy. However, clinical events such as cancer recurrence, clinical deterioration, unplanned hospitalization or readmission, and treatment changes were not prospectively or systematically collected as study safety outcomes during the inpatient period or post-discharge follow-up.

## 4 Discussion

In this randomized controlled trial, a five-session group-based ACT program reduced FoP and psychological distress in young and middle-aged patients with breast cancer receiving particle therapy. Psychological flexibility also improved, although the between-group effect was evident only at the final follow-up. These findings provide evidence for delivering ACT during active particle therapy, when patients are managing treatment procedures, toxicities, and uncertainty about disease progression.

The reduction in FoP was consistent with a previous randomized trial of group-based ACT in patients with breast cancer and high FoP (12), in which the intervention produced sustained reductions in fear relative to a control condition. It also accords with meta-analytic findings that ACT reduces fear of recurrence and progression across cancer populations (38). A broader meta-analysis of psychological interventions reported a small pooled effect on fear of recurrence or progression, with larger effects when fear was the primary intervention target and when interventions were delivered in groups (39). The greater reductions observed in the present trial are consistent with these moderators because FoP was the primary outcome and ACT was delivered in a group format. Whereas most previous work has focused on post-treatment survivorship, our findings extend the benefit of ACT to patients actively undergoing particle therapy, a period of intensive daily treatment, high expectations of a precise and low-toxicity modality, and pronounced uncertainty about long-term outcomes. The control group also improved over time, possibly because of psychosocial support within usual care, the passage of time, and regression to the mean. Nevertheless, the significant group × time interactions indicate that ACT provided additional benefit beyond usual care. However, because the control condition did not match the intervention for therapist contact, group interaction, or treatment expectancy, the between-group differences cannot be attributed solely to ACT-specific processes. Some of the observed benefit may have reflected these nonspecific therapeutic factors. Future trials using an attention-matched condition, such as structured psychoeducation or a supportive group intervention, would help distinguish the specific effects of ACT from those associated with attention and group participation.

The magnitude and early onset of improvement may partly reflect the elevated FoP of the study population. Younger age is consistently associated with greater FoP among patients with breast cancer. Younger long-term survivors are substantially more likely to report clinically relevant fear (17), and younger age, lower self-management efficacy, and weaker family support explain a considerable proportion of the variance in FoP (16). Our participants closely matched this high-risk profile. They entered the trial with fear levels well above the clinical threshold. Such severe yet modifiable fear provides substantial room for improvement. This may help explain the large and early within-group reduction observed in the intervention arm.

The observed improvement is also consistent with current understanding of how FoP is maintained. FoP is not a single thought that can simply be removed through rational argument. According to the self-regulatory executive function model, fear of recurrence or progression is sustained by metacognitive beliefs about worry and by coping responses such as persistent worry, threat monitoring, and emotional suppression (40). This is consistent with studies showing that maladaptive metacognition and avoidance-oriented coping are associated with higher FoP (41). Recent qualitative evidence provides a similar picture. Breast cancer survivors commonly report avoidance and emotional detachment, including distraction and hiding concerns, while patterns of worrying in silence and avoiding negative thoughts have also been described in other cancer populations (22, 23). Such responses may provide temporary relief but may not address the underlying fear in a sustained way (22). Experiential avoidance and thought suppression may further intensify psychological symptoms during radiotherapy (42). ACT directly addresses these processes. Acceptance and cognitive defusion can help patients make room for distressing thoughts and reduce attempts to suppress them (30, 43), while present-moment awareness may lessen future-oriented worry (44). By helping patients change their relationship with fear, ACT may support value-based living during the uncertainty of particle therapy.

Improvement in psychological flexibility emerged gradually and became significant only at the final follow-up in both the primary and sensitivity analyses. This trajectory is consistent with the ACT framework, in which patients need repeated practice to move from cognitive fusion and experiential avoidance toward more flexible responses (24). Qin et al. (45) reported that a six-session ACT program for cancer patients receiving proton and heavy ion therapy significantly improved psychological flexibility both immediately after the intervention and at 3-month follow-up. Their findings support the relevance of ACT for enhancing psychological flexibility in the particle therapy setting. The later effect in the present trial may reflect the shorter 2.5-week program and the introduction of values clarification and committed action primarily in the final session. Participants may have first learned to use specific ACT skills to manage progression-related thoughts and treatment stress, before these skills were reflected in a broader self-rated change in psychological flexibility. This interpretation is consistent with findings that increases in valued behavior can precede reductions in suffering (46). However, the present study did not test whether changes in psychological flexibility mediated the effects of ACT on FoP or psychological distress. The delayed improvement in psychological flexibility should therefore be regarded as a plausible process rather than evidence of a confirmed mechanism. Future studies with repeated assessments could use longitudinal mediation analysis or structural equation modeling to examine whether changes in psychological flexibility predict subsequent reductions in FoP and distress. Brief ACT delivered during radiotherapy may therefore benefit from continued homework, nurse reinforcement, or booster contact after discharge to consolidate acceptance- and values-based skills.

The ACT reduced psychological distress at all post-intervention assessments, and the effects remained significant in the sensitivity analysis. This finding is noteworthy because baseline distress was relatively low, leaving limited room for improvement. ACT may have changed how patients responded to distress during treatment. Experiential avoidance and cognitive fusion are associated with greater distress in cancer populations, whereas acceptance and present-moment awareness are associated with lower distress (47). This accords with the view that distress in young and middle-aged breast cancer patients arises less from negative emotions themselves than from experiential avoidance, emotional suppression, and maladaptive appraisal of internal sensations (48). ACT may have helped patients allow difficult internal experiences while continuing valued activities (24, 30). The maintenance of treatment effects through 3-month follow-up, consistent with prior ACT studies in oncology (45), further suggests that these benefits reflect skill internalization and contextual transfer rather than transient symptom control (47). The repeated treatment schedule in particle therapy provides an opportunity to reinforce ACT skills across treatment visits. Oncology nurses may extend support after discharge through structured follow-up, digital reminders, and mini-program-based homework review, helping patients practice acceptance, cognitive defusion, and values-based action in daily life. However, the Distress Thermometer is a single-item screening measure and may not capture more nuanced changes across different dimensions of psychological distress. Future studies could combine the Distress Thermometer with multidimensional measures, such as the Hospital Anxiety and Depression Scale, to provide a more detailed assessment of emotional outcomes.

The exclusively Chinese sample also warrants consideration of cultural influences on ACT delivery and effectiveness. Mental health stigma and cultural norms related to collectivism, interpersonal connectedness, and emotional disclosure may affect both participation and group dynamics (49, 50). Greater emphasis on family and interpersonal relationships may also shape how patients approach values clarification and committed action (51). Future multicenter studies should examine these cultural moderators and whether adaptations to ACT delivery are needed across different populations.

### 4.1 Implications for nursing practice and research

This study has practical implications for oncology nursing. FoP screening at the start of particle therapy, using a brief tool such as the FoP-Q-SF, may help identify patients who need timely psychological support. The five-session group ACT program can be scheduled alongside repeated radiotherapy visits, which may facilitate delivery during active treatment. With appropriate ACT training and clinical supervision, oncology nurses may reinforce between-session practice, monitor distress and FoP during follow-up, and facilitate referral to psycho-oncology services when clinically indicated. Evidence from other radiotherapy settings supports the feasibility of nurse-led supportive care (52), although the feasibility and effectiveness of nurse-led ACT remain to be established. Larger multicenter trials should evaluate nurse-led and digitally supported ACT delivery models.

### 4.2 Limitations

Several limitations should be noted. The single-center design, modest sample size, exclusively Chinese sample, and 3-month follow-up limit the generalizability of the findings across cultural and clinical settings and conclusions regarding the durability of the intervention effects. The use of usual care rather than an attention-matched control limits our ability to distinguish ACT-specific effects from nonspecific effects related to therapist attention, group interaction, and treatment expectancy. Reliance on self-reported outcomes may introduce reporting bias, and the single-item Distress Thermometer may have limited sensitivity to nuanced changes in psychological distress. Validation evidence for the Chinese measures in breast cancer populations also remains limited. The DT Problem List was not administered, and reasons for missed sessions were not systematically recorded, limiting our ability to characterize sources of distress and barriers to participation. Relevant clinical events were not systematically collected as study safety outcomes. The study was not designed to test mediation or dose–response effects, so the role of psychological flexibility as a mechanism and the influence of session attendance remain to be examined. Future multicenter trials with longer follow-up, attention-matched control conditions, prospective collection of process and adherence data, and standardized adverse event monitoring are warranted.

## 5 Conclusion

This randomized controlled trial showed that ACT reduces FoP and psychological distress in young and middle-aged patients with breast cancer receiving particle therapy. Improvement in psychological flexibility emerged later and was evident at the 3-month follow-up. Structured group-based ACT can be delivered within routine radiotherapy care and may provide a practical approach to psychological support during active treatment. These findings support further evaluation of ACT-informed care in particle therapy settings.

## Data Availability

The raw data supporting the conclusions of this article will be made available by the authors, without undue reservation.
